# Tomographic breathing detection: a method to noninvasively assess *in situ* respiratory dynamics

**DOI:** 10.1117/1.JBO.23.5.056011

**Published:** 2018-05-30

**Authors:** Devin O’Kelly, Heling Zhou, Ralph P. Mason

**Affiliations:** University of Texas Southwestern Medical Center, Department of Radiology, Dallas, Texas, United States

**Keywords:** breathing detection, photoacoustics, multispectral optoacoustic tomography, respiratory motion, motion detection

## Abstract

Physiological monitoring is a critical aspect of *in vivo* experimentation, particularly imaging studies. Physiological monitoring facilitates gated acquisition of imaging data and more robust experimental interpretation but has historically required additional instrumentation that may be cumbersome. As frame rates have increased, imaging methods have been able to capture ever more rapid dynamics, passing the Nyquist sampling rate of most physiological processes and allowing the capture of motion, such as breathing. With this transition, image artifacts have also changed their nature; rather than intraframe motion causing blurring and deteriorating resolution, interframe motion does not affect individual frames and may be recovered as useful information from an image time series. We demonstrate a method that takes advantage of interframe movement for detection of gross physiological motion in real-time image sequences. We further demonstrate the ability of the method, dubbed tomographic breathing detection to quantify the dynamics of respiration, allowing the capture of respiratory information pertinent to anesthetic depth monitoring. Our example uses multispectral optoacoustic tomography, but it will be widely relevant to other technologies.

## 1 Introduction

During *in vivo* experiments, it is desirable to develop an understanding of the subject’s systemic physiology, to control for experimental aberrations and to better interpret experimental results. Physiological information may be particularly relevant to imaging investigations. In magnetic resonance imaging (MRI), for example, physiological monitoring enables the acquisition of cardiac- and respiratory-gated images, enabling better interimage correlations that are not corrupted by motion. Tomographic imaging is a broad designation given to any method that provides a cross-sectional slice of the imaged subject, whether it is material, human, or animal. As the hardware for tomographic imaging systems has improved, so too has the time resolution of image acquisition, such that interimage movement is often substantially greater than intraimage movement.^1^ At the same time, computational resources have improved such that real-time image reconstruction can be performed even using model-based methods.^2^ This capability allows the imaging system access to a train of reconstructed images in real time. In realistic *in vivo* scenarios, some proportion of these images will be displaced relative to others as a result of motion caused by physiological processes, e.g., breathing, as noted in several previous publications.^3^^–^^6^ Online averaging of images “smooths out” this displacement^7^ but compromises resolution in the process. This is due to the fact that averaging is ostensibly intended to improve signal-to-noise ratio, according to the central limit theorem (CLT). However, one of the central assumptions in the CLT is that the distribution is stationary, which is clearly not the case for a dynamic image. If some processing takes place before averaging, however, we may derive useful information regarding physiological motion. This motion information has a benefit of being presynchronized to the image time series. We demonstrate the concept in terms of respiratory motion observed in freely breathing anesthetized mice undergoing photoacoustic imaging during a gas breathing challenge.

## 2 Methods

### 2.1 Algorithm

**Fig. 1:**
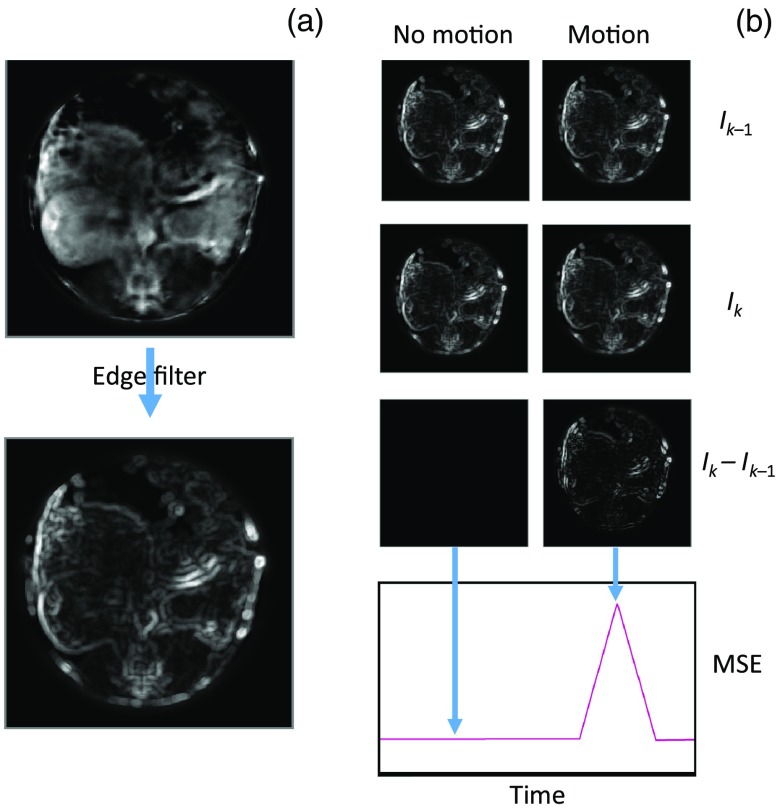
Cartoon overview of the method: (a) an image series (e.g., Video 1, MP4, 129 kB [URL: http://dx.doi.org/10.1117/1.JBO.23.5.056011.1]) is bandpass filtered and (b) the MSE between each sequential image is calculated. This error may then be used to infer the presence of motion between a given pair of frames.

The ability to correct for physiological motion is contingent upon identifying it. Identifying differences between image frames is a well-studied topic in the field of motion estimation and video compression.^8^ We have chosen to implement a filter-based approach (shown in Fig. 1, details in Appendix A) due to its ease of implementation and low computational overhead. In brief, the method bandpass filters two sequential images to remove low-frequency contrast information and noise, giving images primarily weighted toward edge contrast. We then apply a box filter to reduce sensitivity to minor motion and calculate the mean squared error (MSE) of the two images as

$$\mathrm{MSE}=\frac{1}{N}\underset{i,j}{\sum }{\left(I_{k}-I_{k-1}\right)}^{2},$$

where N is the number of pixels in a given image, $I_{k}$ is the current filtered image, and $I_{k-1}$ is the previous filtered image. MSE provides a measure of the degree of movement across the entire imaging region. A fundamental assumption is that the image contrast changes relatively slowly from frame to frame, while edge motion is guided by more rapid dynamics. This is reasonable in many scenarios, including contrast agent infusion; as large areas of contrast change do not affect the MSE due to the initial high-pass step, any contrast change will be in a relatively localized area and is unlikely to contribute substantially to the MSE over the entire image plane.

There are many varieties of physiological motion which degrade image quality and resolution, including respiratory,^5^ cardiac,^6^ and subject^4^ movement. In most *in vivo* imaging scenarios, suppression or compensation of this motion is rather challenging. By tracking MSE over time, however, we may take advantage of this motion to derive physiological information without any additional instrumentation. In this study, we use respiratory motion as an example, due to its approximate periodicity and dominance in contributing to overall body motion as compared to the relatively minor effects of cardiac motion.

Given a time course of the MSE between images, we would like to recover information about breathing rates and breathing dynamics. To recover the breathing rate, we must perform some form of time-frequency analysis, to determine the breathing rate at each point in time. This might be achieved by determining the time between individual peaks, but this approach is fundamentally discontinuous and does not reflect our assumption that the modulation of breathing dynamics is a continuous process. We have, therefore, chosen to use wavelet analysis to preserve both time and frequency continuity.^9^

We first subtract the mean of the pulse train and then apply a synchrosqueezed wavelet transform (SSWT).^10^ The SSWT effectively performs a standard one-dimensional wavelet transform followed by localization in frequency, giving higher time-frequency resolution than other methods. We may then apply ridge detection to the resultant transform, recovering the primary mode of the time series, which should correspond to the instantaneous breathing rate at each point in time. This instantaneous breathing rate, in turn, is time-continuous and may be analyzed further to examine the stability of the breathing rate, for example, with respect to anesthetic or gas monitoring.^11^

To accommodate periods of time when there may be lapses in breathing, we note that the absence of a breathing signal will correspond to lower total signal energy. If we threshold based on this energy, then absence of breathing will be detected whenever the total signal energy drops below the threshold. We may improve robustness further by tracking the amount of energy “around” the detected time-frequency ridge, which will provide some amount of noise rejection. The details of this approach are given in Appendix B.

A goal is to achieve a stable baseline from which variation due to experimental interventions may be compared. In experiments performed under anesthesia, it is critical that the physiological state induced by a certain amount of anesthetic is sufficiently stable so as to not introduce artifacts in experiments.^12^ The monitoring of anesthetic depth is frequently assessed using a combination of signals ranging from reflex response^13^ to high-order statistical analysis of electroencephalography data in the form of the bispectral index,^14^^,^^15^ but most of these methods require specific instrumentation or contact intervention.

We use an imaging modality known as multispectral optoacoustic tomography (MSOT)^16^^,^^17^ as an example of tomographic imaging. MSOT illuminates an imaging target with wavelength-selectable near-infrared light in pulses of duration on the order of nanoseconds. This achieves stress confinement, inducing local thermal expansion, and causing ultrasound pressure waves to propagate from the point of absorption. These waves are then detected by a circular transducer array surrounding the target, providing data which may then be reconstructed into an image coded by the selected wavelength of light.

### 2.2 Experimental Verification

All animal work was approved by the institutional animal care and use committee at the University of Texas Southwestern in accordance with United States federal guidelines.

MSOT data were acquired using an InVision 256-TF (iThera Gmbh, Munich, Germany). The description of detailed technical specifications is available elsewhere.^16^ Briefly, the imaged subject is coated in ultrasound gel, wrapped in a polyethylene film, and placed in a holder, which allows the subject to rest in a heated water bath. Wavelength-specific laser pulses then illuminate the subject, causing photoacoustic thermal expansion, which induces pressure waves that are then detected by circumferentially arranged ultrasound transducers.

**Fig. 2:**
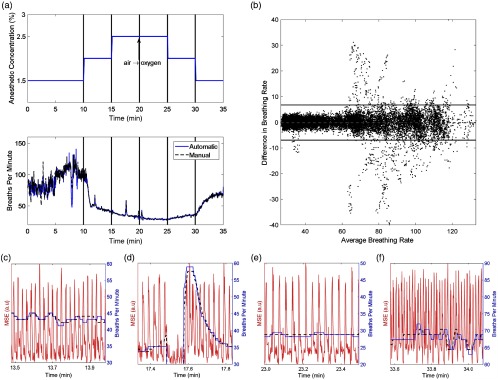
(a, upper) Experimental protocol for isoflurane challenge. A mouse was induced while breathing 1.5% isoflurane in air at 2  L/min for 20 min and then underwent the isoflurane and oxygen challenge experiment. The animal breathed air for the first 20 min, at which point the breathing gas was switched to oxygen. (a, lower) Breathing rate determined from the isoflurane challenge experiment with both the MSE method and the manually annotated data. (b) Bland–Altman plot showing the variation in detected breathing rate between the MSE and manual breathing traces. The differences between the methods have a mean of 0.0±3.3  bpm. (c–f) Time courses of MSE and recovered breathing rate under (c) 2.0%, (d, e) 2.5%, and (f) 1.5% isoflurane exposure, while breathing (c, d) air and (e, f) oxygen. In (d), we see the capture of a breathing transient in compensation for suspended breathing, whereas (f) shows the increased breathing rate due to switching back to 1.5% isoflurane. Note the change of scale in (e, f).

Specifically, an anesthetized female nude mouse in a supine position was imaged cross sectionally at the level of the kidneys and liver in a water bath at 35°C. This area has both rich structure and substantial diaphragmatic motion resulting from breathing. Data were collected at wavelengths ranging from 700 to 860 nm, in steps of 20 nm. Four laser shots were acquired per wavelength, unaveraged at a rate of 10 Hz. This wavelength set was then acquired repeatedly for the entire imaging session. Images of a 2.5-cm field of view were reconstructed using ViewMSOT^®^ v3.6 software (iThera Gmbh, Munich, Germany) to a resolution of 75  μm. Data were collected from a mouse breathing isoflurane and medical air or 100% oxygen [Fig. 2(a), upper]. After a preliminary imaging session of ∼20  min to acclimate the mouse to the water bath temperature and warm up the device’s laser, the level of anesthetic was varied to induce changes in breathing rate under several anesthetic conditions (1.5%, 2%, and 2.5% isoflurane) in one continuous scan over a period of 35 min. The breathing gas was switched from air to oxygen at 20 min to observe any substantial variations in breathing rate due to the ${\mathrm{pO}}_{2}$ of inspired gas.^18^ Reconstructed image data were then processed using methods available from the Image Processing and Wavelet Toolboxes in MATLAB^®^ (The MathWorks Inc.).

Objective validation of the quantified breathing rate was performed by manual tagging of breathing events in the image time series. These manually registered tags were then gridded via nearest-neighbor interpolation and smoothed by convolution with a triangular kernel to lessen harmonic interference. The rectified dataset then underwent the same time-frequency analysis as the MSE data.

## 3 Results

When applied to the breathing video (Video 1), tomographic breathing detection (TBD) was able to extract respiratory motion with high reliability [Fig. 2(a), lower]. After SSWT analysis, the method was able to quantitatively return the breathing rate over a wide range of respiratory behaviors ranging from highly regular, consistently spaced breathing [Figs. 2(c), 2(e), and 2(f)] to intermittent, irregular breathing [Fig. 2(d)], and at a range of breathing rates. Interestingly, the change to oxygen at 20 min was not seen to dramatically increase respiratory activity, in contrast with previous observations.^18^ We attribute this to the depth of anesthesia at the time of gas change, though other explanations, such as systemic ${\mathrm{CO}}_{2}$ accumulation,^19^ are possible.

Due to the ridge-finding algorithm’s presumption of continuity, instances when the mouse ceased breathing for extended periods introduced artifacts of spuriously high or low breathing rates. In practice, however, these rates would be easily identified by masking the breathing rate based on the energy thresholding approach described in Appendix B.

## 4 Discussion

We have demonstrated a method to recover breathing dynamics from imaging sequences with high temporal resolution, even in scenarios with changing illumination and contrast. The periodic respiratory motion is readily recoverable through a simple algorithm and yields breathing information that strongly recapitulates objectively validated data.

**Fig. 3:**
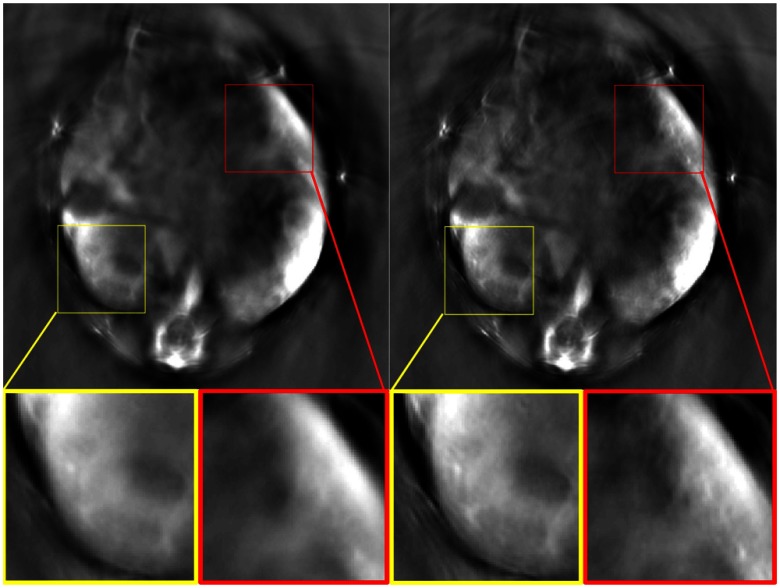
Comparison of averaged images (a) without (N=32) and (b) with (N=12) removal of image frames exceeding the first quartile of the MSE distribution. Improved resolution due to decreased blurring may clearly be seen, particularly in regions with rich vascular structure (insets). The images are pooled from the 800-nm channel of the first 300 image frames of Video 1.

The method, as presented, is quite rudimentary yet captures a substantial amount of information pertaining to respiratory physiology. Extensions are manifold, ranging from more sophisticated edge filtering to online SSWT analysis,^20^ any of which could substantially improve practicality without necessarily adding to the difficulty of implementation. We implemented the method in the image domain, although most imaging methods acquire their data in some other signal domain. Nevertheless, similar processing may take place in the signal domain by comparing variation between datasets. Additional processing, such as retrospective clustering or dynamic resampling of high-motion frames, could be performed using the MSE signal as a feature, allowing for lower-complexity clustering problems. The MSE signal may also be used as a criterion for motion correction, by removing high-MSE images from the dataset which is then averaged, as shown in Fig. 3.

TBD is context agnostic and could be applied to MRI, CT, ultrasound, or any other imaging modality that provides tomographic images, including other, three-dimensional implementations of photoacoustic imaging,^21^ particularly those which record data at near-real-time rates.^22^ The application of the SSWT for the purposes of breathing detection may be applied to any data that capture the motion of the chest wall, such as laser range-finding or pressure transducers coupled to the imaging water bath.^5^ Notably, the process is quite flexible and requires relatively little parameter tuning; many different image filters accomplish the task of recovering a suitable MSE signal. Furthermore, the method preserves locality; many other methods correlate images across an entire dataset to provide motion correction,^5^^,^^6^ which is unsuitable for imaging dynamic effects, though some methods have adopted other sophisticated processing methods to improve spatiotemporal resolution.^22^

The method has additional application to anesthetic depth monitoring. A classification of anesthetic depth was provided by Thomas et al.,^13^ Bhargava et al.,^14^ and Guedel et al.^23^ and separates progression of anesthetic effect into four planes. Of particular note is that plane II is a highly chaotic state providing a poor baseline, characterized by irregular breathing and a generally unstable breathing rate, whereas breathing in plane III is much more stable and regular and, therefore, preferable for experiments, especially those which probe oxygenation status as an experimental covariate.^12^

The effects of varying anesthesia in the isoflurane challenge as shown in Fig. 2 are notable: the transition from 1.5% to 2.0% isoflurane is marked by a substantial drop in mean respiratory rate, as well as an increase in regularity. This likely corresponds to the animal achieving a deeper plane of anesthesia, transitioning from planes II to III. Even within plane III, variations in breathing rate in response to changes in isoflurane concentration may be noted, particularly at the 25-min mark. The transition from planes III to II, i.e., to a more mild state of anesthesia, is seen at 30 min after switching to 1.5% isoflurane. The ability to track animal respiration, potentially in real time, opens possibilities for more standardized experimental protocols, as well as accounting for varying anesthetic sensitivity between groups and individuals. Moreover, by forming a quantitative signal of respiratory rate, a feedback system could be developed, which varies the level of anesthetic delivered to a subject to achieve a given respiratory rate.

## Appendix A: Details of Image Analysis

Images are first high-pass filtered to remove contrast information, as two consecutive images may be taken with different contrast parameters/wavelengths without having substantial motion.

These edge images are then filtered with a blurring/low-pass kernel to perform rudimentary denoising, as well as to smooth the calculation of the MSE. This filtering is given by the following equation:

$$I_{\mathrm{filt}}=(I⊗h_{h})⊗h_{l},$$

where $h_{h}$ and $h_{l}$ are the arbitrary high- and low-pass filters, respectively. These filters may be combined into a bandpass filter or left separate in cases of nonlinearity (e.g., using a median filter as the low-pass filter). In this study, we simply used MATLAB^®^’s default “unsharp” filter for high pass and the median filter for low pass, though we found a number of different combinations to be effective.

The point-wise difference of the sequential images is taken as

$$I_{d}=I_{\mathrm{filt}}[k]-I_{\mathrm{filt}}[k-1].$$

By high-pass filtering the images, we generate a representation that is insensitive to contrast but retains edge definition. We then take the difference of consecutive high-passed images, generating a correspondence image. This correspondence image should be near-zero if the images are well-registered and, similarly, should have some mismatch across its field of view if they are not. From there, we may calculate the MSE of the given image in relation to its predecessor

$$\mathrm{MSE}[k]=\frac{1}{N}\underset{i,j}{\sum }{\left(I_{d}[k]\right)}^{2}.$$

This process may then be repeated over a dataset or as data arrives from the imaging procedure. Pairs of images with low MSE will correspond to images that are relatively well-aligned, while pairs of images with high MSE will correspond to images where there is some gross motion.

## Appendix B: Energy Thresholding

Given an MSE trace for all time indices k, as in Appendix A, we perform an SSWT to return a time-frequency image with time index k and frequency bin ν

W[k,ν]=SSWT(MSE[k]).

Then applying a ridge-finding algorithm will return the dominant signal trace given as

N[k]=RidgeFind(W[ν,k]).

Locally summing the energy around this trace can be accomplished by taking the sum of absolute values in the time-frequency bins around the ridge

$$E[k]=\overset{J}{\underset{j=-J}{\sum }}|W[k,N[k]+j]|,$$

where J is an integer number, J=5 in this study, though the number of local bins depends on the time-frequency resolution chosen in the wavelet transform. This summed value then provides an energy term that may be thresholded to create a mask of “valid” breathing rates

$$N_{\text{valid}}[k]=\{N[k]|E[k]\geq \tau \}.$$

We acknowledge that the precise value of τ is subject to some degree of tuning and is dependent on the nature of the image and signal statistics. Automatic determination of this threshold may be a fruitful direction of future investigation.
